# Spectral sensing at a glance

**DOI:** 10.1038/s41467-026-78586-7

**Published:** 2026-10-14

**Authors:** Andreas Tittl

**Affiliations:** Institute of Photonics, Hamburg University of Technology, Hamburg, Germany

**Keywords:** Optical sensors, Biosensors

## Abstract

Portable biosensors are revolutionizing the way we diagnose disease and ill-health. By varying a metasurface’s geometry continuously across a chip, a recent study by H. Kim et al. reports a new sensing strategy, which reads signals from individual camera images without the need for a spectrometer. This enables the label-free detection of biomarkers in a handheld format.

Optical biosensors built from resonant nanostructures have traditionally faced a stubborn trade-off. On one hand, metasurface sensors supporting bound states in the continuum (BICs)^1^ target high quality-factor resonances, whose narrow linewidths make small spectral shifts easier to resolve. On the other hand, their narrow linewidths demand high-resolution spectrometers to resolve the tiny wavelength shifts associated with molecular binding. Thus, these otherwise compact sensors still often require bulky, costly instrumentation, holding back their transition towards point-of-care applications. Illumination with single-wavelength sources can remove the need for spectrometers^2^, but the use of high-Q resonances can constrain the operational spectral range, which limits the dynamic range of the detection.

Kim and colleagues overcome these limitations by building a refractive index sensor in the visible spectrum that encodes the target wavelengths in space^3^. Across a 300-µm chip, they vary the geometry of an amorphous-silicon unit cell supporting a quasi-BIC resonance continuously so that each column resonates at a slightly different wavelength. Under monochromatic illumination from a compact laser diode, only the unit cells matching the source “go dark”, printing a sharp line across the transmission image. When binding raises the local refractive index, the resonance red-shifts and the line slides sideways (Fig. 1). By reading the output against a calibrated map, its position reports the spectral shift directly.

**Fig. 1:**
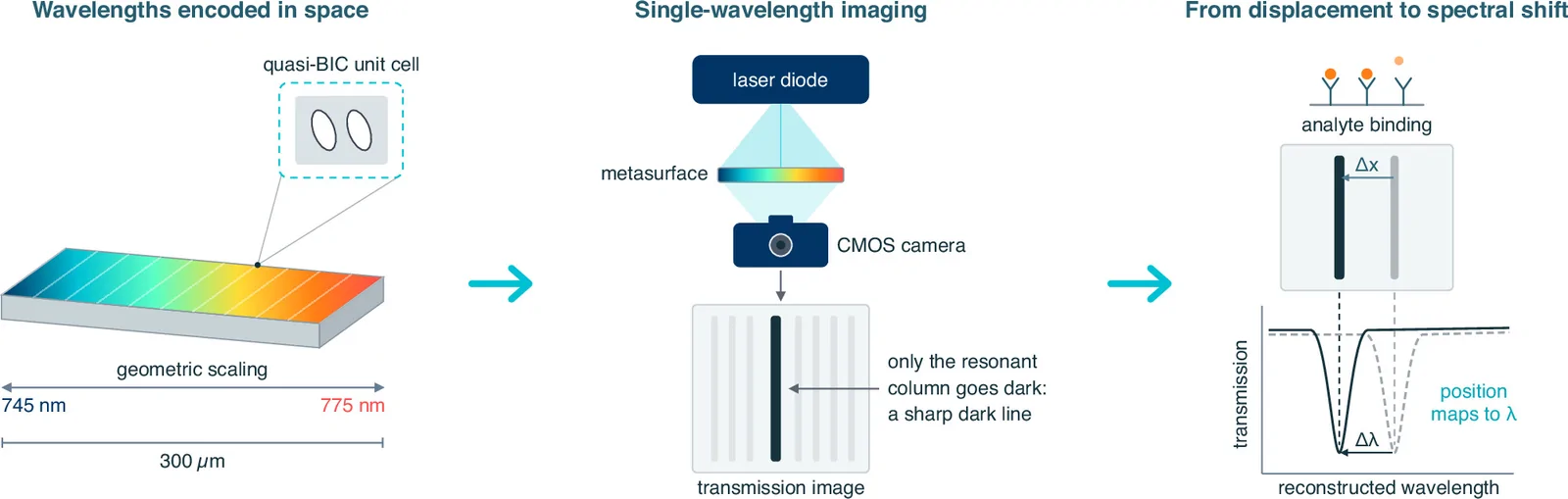
Spectral sensing from single images. In a gradient metasurface, a quasi-BIC unit cell is scaled continuously across the chip, mapping a distinct resonance wavelength to each lateral position (color gradient). Under monochromatic illumination, the resonant column appears as a dark line in the camera image. Molecular binding raises the local refractive index, shifting the resonances and displacing the line. Its calibrated position yields the resonance shift and a reconstructed spectrum without a spectrometer.

Because adjacent cells differ only slightly, the array behaves locally as though uniform, preserving a high Q (~345) while delivering a quasi-continuous spectrum across a broad 30-nm range. In fact, the spatial gradient decouples spectral coverage from resonance linewidth so that each local resonance remains narrow, while the metasurface as a whole spans a much broader wavelength range. The device resolves 0.1-nm shifts, reaches a figure of merit of 67.2 per refractive index unit, and, coupled to a fingertip-sized diode-and-camera module, detects streptavidin down to a few hundred picomolar concentrations, all completely label-free.

The unique appeal of this approach is that it transforms the sensitivity-versus-resolution trade-off into a matter of spatial layout, effectively imprinting a spectrometer onto the metasurface itself. Some challenges remain: the achievable Q is capped by fabrication imperfections, and the quasi-BIC weakens near the device edges, potentially limiting the usable sensing area. More generally, spatial encoding transfers the requirement for spectral precision to positional precision, making accurate calibration and image registration important for practical device deployment. With scalable patterning such as nanoimprint or deep-ultraviolet lithography, and with different regions functionalized for parallel readout of wavelengths and potentially even Q factors^4^, the concept is uniquely positioned to deliver multiplexed chips that could carry high-Q nanophotonic sensing from the laboratory into the field.

## Data Availability

No datasets were generated or analyzed for this article.
